# The Prognostic Role and Relationship between E2F1 and SV40 in Diffuse Large B-Cell Lymphoma of Egyptian Patients

**DOI:** 10.1155/2015/919834

**Published:** 2015-10-27

**Authors:** Rehab M. Samaka, Hayam A. Aiad, Mona A. Kandil, Nancy Y. Asaad, Nanes S. Holah

**Affiliations:** Pathology Department, Faculty of Medicine, Menoufia University, Shebin El-Kom, Egypt

## Abstract

Diffuse large B-cell lymphoma (DLBCL) is the most common type of lymphomas worldwide. The pathogenesis of lymphomas is not yet well understood. SV40 induces malignant transformation by the large T-antigen (L-TAG) and promotes transformation by binding and inactivating p53 and pRb. L-TAG can bind pRb promoting the activation of the E2F1 transcription factor, thus inducing the expression of genes required for the entry to the S phase and leading to cell transformation. This immunohistochemical study was conducted to assess the prognostic role and relationship of SV40 L-TAG and E2F1 in diffuse large B-cell lymphoma (DLBCL) of Egyptian patients. This retrospective study was conducted on 105 tissue specimens including 20 follicular hyperplasia and 85 DLBCL cases. SV40 L-TAG was identified in 3/85 (4%) of DLBCL. High Ki-67 labeling index (Ki-67 LI) and apoptotic count were associated with high E2F1 expression (*p*<0.001 for all). No significant association was reached between E2F1 and SV40. E2F1 expression proved to be the most and first independent prognostic factor on overall survival of DLBCL patients (HR = 5.79, 95% CI = 2.3–14.6, and *p*<0.001). Upregulation of E2F1 has been implicated in oncogenesis, prognosis, and prediction of therapeutic response but is not seemingly to have a relationship with the accused SV40.

## 1. Introduction

Non-Hodgkin's lymphoma (NHL) is the third common malignancy out of all malignances of Egyptian patients; it is of high rank among cancers in each sex, where it accounts for 8.4% of estimated incidence with 8.7 age standardized rate (ASR) per 100.000 [1]. In Egypt, NHL represents a major health problem as its rates are one of the highest in the world [2]. Diffuse large B-cell lymphoma (DLBCL) is the most common lymphoma worldwide [1].

Alarmingly, Simian virus (SV40) induces malignant transformation in rodents and human cells. This transformation is induced by the large T-antigen (L-TAG), known to promote transformation by binding and inactivating tumor suppressor genes, such as p53 and pRb [3]. Over the years, an increasing number of reports have suggested that SV40 causes specific tumor types, such as mesothelioma, brain, and bone tumors [4]. L-TAG can bind pRb promoting the activation of the E2F1 transcription factor, thus inducing the expression of genes required for the entry to the S phase [3]. Few studies have shown the presence of SV40 in lymphomas [4]. Nevertheless, SV40 could be taken into consideration for a putative role in human lymphomagenesis, alone or in combination with additional events, such as a transcription factor E2F1.

E2F is a family of transcription factors that regulate the expression of genes involved in a wide range of cellular processes, including cell-cycle progression, DNA repair, differentiation, and apoptosis [5]. E2F1, the founding member of the family, induces proliferation; both Rb-deficiency and ectopic expression of E2F1 in normal cells lead to high level of apoptosis owing to its ability to activate a large number of proapoptotic genes through a plethora of distinct apoptotic mechanisms [6]. However, the information about the role of E2F1 in human malignancy as depicted from its expression in relationship to tumor kinetic parameters and clinicopathological features is limited and incomplete.

The present immunohistochemical (IHC) study was conducted to assess the role and relationship of SV40 L-TAG and E2F1 in DLBCL of Egyptian patients, get a hint of whether SV40 and E2F1 are coplayers in this malignancy or not, and correlate the results with the standard clinicopathological and survival data.

## 2. Materials and Methods

### 2.1. Studied Population

This retrospective case control study was conducted on 105 archival cases, including 85 DLBCL cases and 20 reactive follicular hyperplasia cases that were used as a control group. They were diagnosed in Pathology Department, Faculty of Medicine, Menoufia University, between January 2003 and December 2007. Written consent forms approved by The Committee of Human Rights in Research in Menoufia University were obtained from studied cases and control subjects before study initiation. Cases were newly diagnosed with no previous treatment taken.

### 2.2. Clinical Features

Staging was evaluated according to Ann-Arbor staging system and then the cases were divided into an early stage, by lumping stages I and II of the tumor, and an advanced stage, by lumping stages III and IV of the tumor. Revised international prognostic index (R-IPI) was calculated and the final scores stratified the DLBCL patients into 3 distinct prognostic groups [7]. For statistical purpose this score was simplified as 0–2 indicating good R-IPI and 3–5 indicating poor R-IPI. Age adjusted IPI (AAIPI) was applied separately in patients younger than or equal to 60 years (AAIPI < 60) and those older than 60 years (AAIPI > 60) to identify 3 risk groups for each category [8].

### 2.3. Histopathological Features

The hematoxylin and eosin (H&E) stained sections were evaluated for the presence and percentage of spontaneous coagulative tumor necrosis. Mitotic and apoptotic figures were counted in 10 randomly selected cellular fields under high power magnification (×400) and they were used as dichotomous covariant in the statistical analysis according to the median value for apoptosis and 25 for mitotic count [9]. The Ki-67 labeling index (Ki-67 LI) was determined using a semiquantitative visual approach and expressed as the percentage of Ki-67 positive malignant cells among a total number of 1000 malignant cells, at high power magnification [10]. Fifty percent cutoff point was applied to discriminate between low and high Ki-67 LI [11]. Scoring was carried out using an Olympus CH2 light microscope (Tokyo, Japan) with a wide angle (field size of 0.274 mm^2^ and field diameter of 0.59 mm^2^).

### 2.4. SV40 L-TAG and E2F1 Immunostaining Procedure

Five-micrometer-thick sections were cut from the paraffin-embedded blocks, deparaffinized in xylene, and rehydrated in a graded alcohol series. Epitope retrieval: the preferred method for SV40 is the use of Heat Induced Epitope Retrieval (HIER) techniques using Cell Marque's Trilogy (Cat. number 920P-04, Cell Marque, 6600 Sierra College Boulevard, Rocklin, CA 95677, USA) and followed by cooling at room temperature. For E2F1 epitope retrieval the tissue sections are boiled in 1 mM EDTA, pH 8.0. The slides were incubated overnight at 4°C with mouse monoclonal SV40 L-TAG Ab-2 with 1 : 100 as optimal dilution (Cat. number 351-14, Cell Marque, 6600 Sierra College Boulevard, Rocklin, CA 95677, USA). Positive control slides of SV40 infected renal tissues were used (Cat. number 351S, Thermo Scientific, Lab Vision Corporation, 46360 Fremont Boulevard, Fremont, CA 94538-6406, USA). The slides were incubated over night at 4°C with mouse monoclonal E2F1 with 1 : 200 as optimal dilution (Cat. number MS-879-P0Ab-2, Thermo Scientific, Lab Vision Corporation, 46360 Fremont Boulevard, Fremont, CA 94538-6406, USA). Breast carcinoma was used as a positive control. The detection kit used was ultravision detection system antipolyvalent HRP/DAB (ready to use) (Cat. number TP-015-HD, Thermo Scientific, Lab Vision Corporation, 46360 Fremont Boulevard, Fremont, CA 94538-6406, USA). The reaction was visualized by an appropriate substrate/chromogen (Diaminobenzidine, DAB) reagent with Mayer haematoxylin as a counterstain.

### 2.5. Assessment of SV40 and E2F1 Immunostained Slides

Positive SV40 expression is assigned when any number of cells shows true nuclear staining regardless of absence or presence of concomitant cytoplasmic staining while only cytoplasmic staining does not assign any positivity [12]. Evaluation of E2F-1 expression is based on the proportion of labeled nuclei either low E2F1 expression (≤10%) or high expression (>10%) [13]. Unintentional bias was prevented by coding patient tissue samples so that IHC analysis was done without knowledge of the patients' outcome and tumor characteristics. Assessment of slides was done by two of the authors (Rehab M. Samaka and Nanes S. Holah) separately.

### 2.6. Statistical Analysis

Statistical analysis was performed using SPSS “Statistical Package for the Social Science” program for windows, version 17, SPSS, Inc., Chicago, Illinois, USA. All factors were used as dichotomous covariates in the statistical analysis. To test whether these variables differed according to clinicopathological parameters and biological markers, the Fisher exact (FE), *χ* ^2^ test, Mann-Whitney test, and Student's *t*-test were used. Log-rank and Cox regression analysis were used for life-table assessment. All *p* values were two-sided; *p* values of <0.05 were considered statistically significant. Kaplan-Meier plots and hazard function curves were used to visualize the survival distribution.

## 3. Results

**Figure 1:**
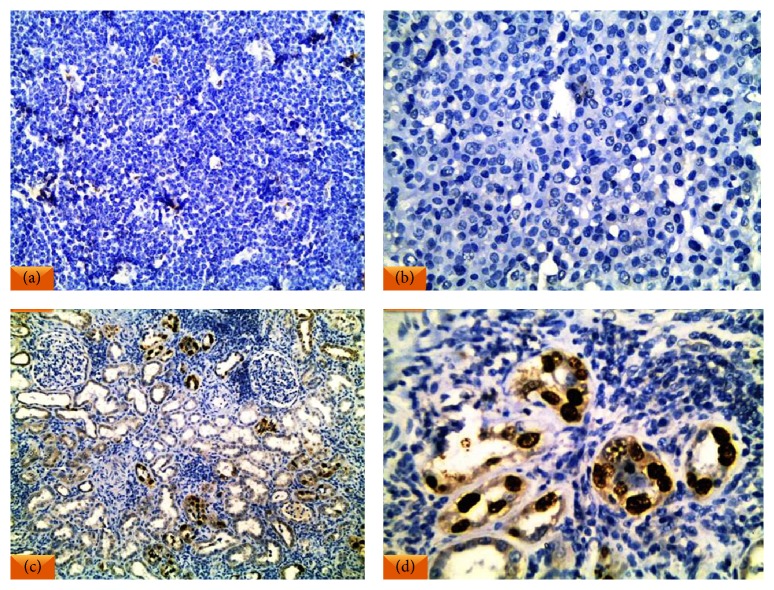
(a) Reactive follicular hyperplasia with prominent tangible body macrophages showed SV40 negativity. (b) Diffuse large B-cell lymphoma (DLBCL) displayed SV40 negativity. (c) SV40 infected renal tissue was the positive control. (d) High power view demonstrating the nuclear SV40 positivity in the tubular cells (IHC ×200 for (a) and (c) and ×400 for (b) and (d)).

**Figure 2:**
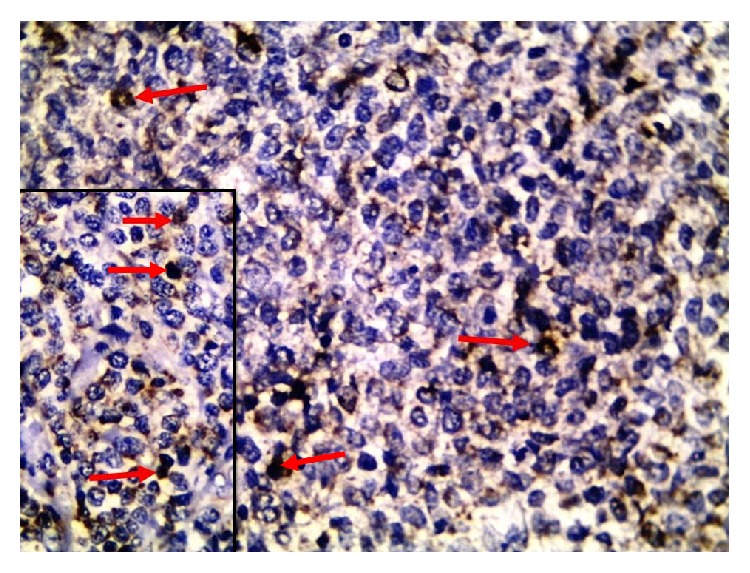
The malignant lymphocytes showed positive nuclear staining (arrows) of SV40 in a case of DLBCL. Inset closer view of nuclear staining of SV40 (arrows) (IHC ×400).

**Figure 3:**
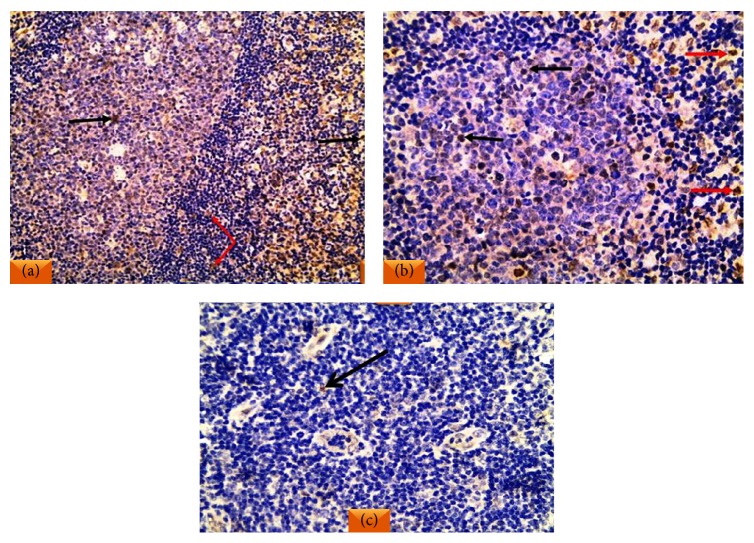
(a) A case of reactive follicular hyperplasia showed positive nuclear staining of E2F1 in the lymphocytes in the germinal center (black arrows) and in the interfollicular area (red arrows). (b) High power view of lymphocytes with nuclear positivity in the germinal center (black arrows) and in the interfollicular area (red arrows) together with negative mantle zone lymphocytes. (c) A germinal center exhibited single lymphocyte with nuclear E2F1 positivity (arrow) (IHC ×200 for (a) and ×400 (b) and (c)).

**Figure 4:**
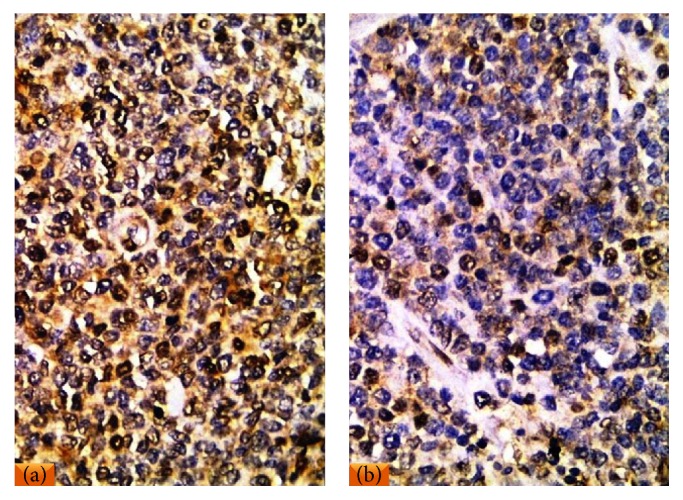
(a) A case of DLBCL showed high nuclear E2F1 expression. (b) A case of DLBCL displayed low nuclear E2F1 expression (IHC ×400 for (a) and (b)).

**Figure 5:**
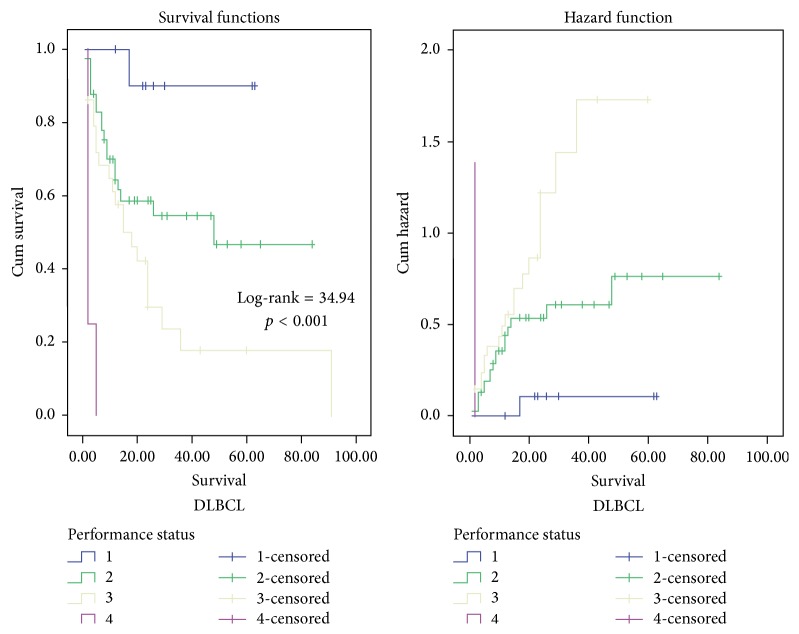
Kaplan-Meier and hazard function curve of overall survival (OS) for DLBCL patients with different categories of performance status (PS) indicating that patients with PS = 4 were more hazardous.

**Figure 6:**
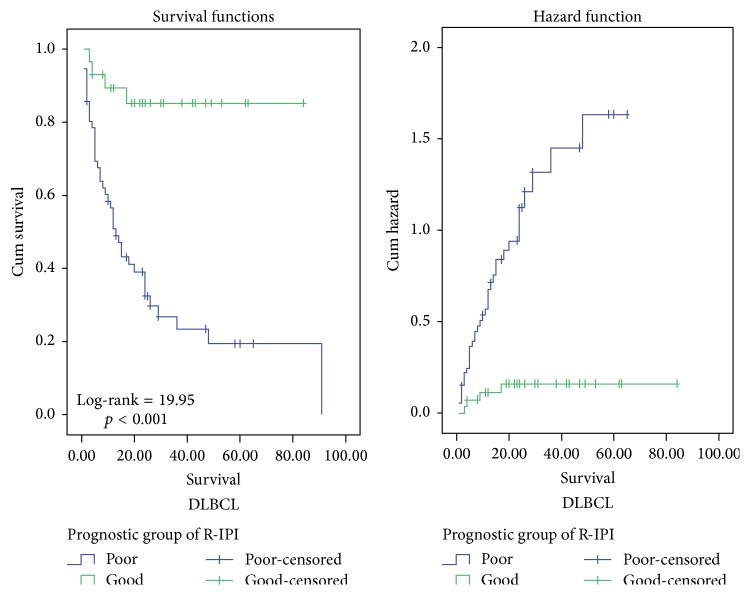
Kaplan-Meier and hazard function curve of OS for DLBCL patients with different categories of prognostic group of R-IPI indicating that poor prognostic group was more hazardous.

**Figure 7:**
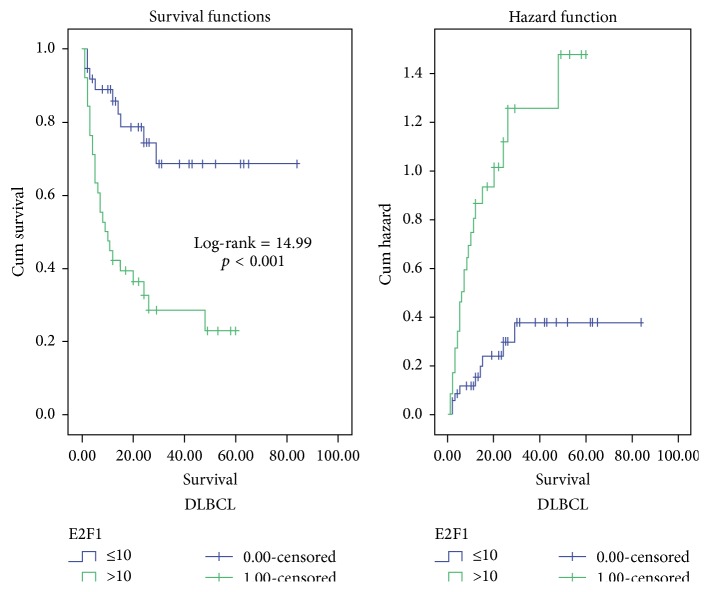
Kaplan-Meier and hazard function curve of OS for DLBCL patients with low and high E2F1 expression indicating that high E2F1 expression was more hazardous.

(i) Clinicopathological data of DLBCL cases studied are shown in Table 1.
(ii) SV40 expression in reactive lymphoid hyperplasia and DLBCL cases is as follows.
  (a) Negative expression of SV40 was noted in all reactive lymphoid hyperplasias (Figure 1(a)). Nuclear positivity for SV40 was identified in only 4% of DLBCL cases (3/85) (Figure 2).
  (b) The profiles of SV40 positive and negative DLBCL cases are shown in Table 2.
(iii) E2F1 expression in reactive follicular hyperplasia and DLBCL cases is as follows.
  (a) All reactive follicular hyperplasia cases showed nuclear E2F1 staining with variable percentages of positivity. The topography of positive lymphocytes was distributed in the germinal centers and in the interfollicular areas with complete negativity in the mantle zone lymphocytes (Figure 3). Low E2F1 expression (≤10%) was detected in 15/20 cases (75%), while high E2F1 expression (>10%) was detected in 5/20 (25%) of them.
  (b) All DLBCL cases studied showed positive E2F1 expression. Regarding DLBCL cases, high E2F1 expression (>10%) (Figure 4(a)) presented in 44/85 of cases (52%), while low E2F1 expression (≤10%) (Figure 4(b)) presented in 41/85 of cases (48%).
(iv) Relationship of E2F1 expression in DLBCL cases studied with the clinicopathological features and presence of SV40 is shown in Table 3.
   There was a highly significant difference between low and high E2F1 expression in DLBCL cases regarding the age and age grouping as the lower numerical values of age had associated with high E2F1 expression (*p* = 0.001 and *p* = 0.02, resp.). Numerous mitoses, high Ki-67 LI, and an abundant number of apoptotic counts were significantly associated with DLBCL cases with high E2F1 expression (*p* < 0.001 for all). There was a significant difference between low and high E2F1 expression in DLBCL cases regarding the risk groups of AAIPI < 60, as 71% of cases with high risk group had high E2F1 expression (*p* = 0.049). There was no significant association between E2F1 expression and presence of SV40 in DLBCL cases.
(v) Survival analysis of DLBCL cases showed the following.
   By univariate survival analysis, ≥60 years age group (log-rank (LR) test = 4.21, *p* = 0.04), worse PS (LR test = 34.94, *p* < 0.001) (Figure 5), elevated LDH (LR test = 4.15, *p* = 0.042), presence of B symptoms (LR test = 4.9, *p* = 0.027), advanced stage (LR = 12.19, *p* < 0.001), poor prognostic group of R-IPI (LR test = 19.95, *p* < 0.001) (Figure 6), high risk group of AAIPI < 60 (LR test = 15.01, *p* < 0.001), high Ki-67 LI and apoptotic counts (LR test = 16.93 and LR test = 12.66, resp., *p* < 0.001 for both), and high E2F1 expression (LR test = 14.99, *p* < 0.001) (Figure 7) had shorter survival time of DLBCL cases. By multivariate survival analysis, E2F1 expression proved to be the most and first independent prognostic factor on overall survival of DLBCL patients (HR = 5.79, 95% CI = 2.3–14.6, and *p* < 0.001).

## 4. Discussion

In Egypt, the high incidence of NHL is possibly related to the exposure of population, at a young age, to various bacterial, parasitic, and viral infections which result in a sustained stimulation of the lymphoid system [1, 14]. In view of limited and controversial data about SV40 in NHL, we decide to explore the prevalence of SV40 in DLBCL tissue specimens of Egyptian patients. In the current IHC study, 3/85 (4%) of DLBCL cases were positive for SV40 L-TAG. It was reported that there is no role of SV40 L-TAG in human lymphomas in patients at risk of having received SV40-contaminated poliomyelitis virus vaccines in Italian, Swiss, and Austrian patients [12]. Also, L-TAG was not detected in a lymphoma series of French and Canadian cases as well as in Spanish patients [15, 16]. Similarly, SV40 L-TAG was detected in 1/25 posttransplant lymphoproliferative disorders and 1/5 AIDS lymphoma in USA [17]. Moreover, weak signals of SV40 L-TAG expression were detected in 12/55 HIV-associated lymphomas in USA and in 4% of Swiss mesothelioma patients [18, 19].

SV40 L-TAG expression in few numbers of DLBCL cases studied might be interpreted by one of the following attributions and theories: (a) absence of the integrated SV40 genome in the host cell and thus absence of permanent expression of the oncoprotein L-TAG [12, 15, 16], (b) the short half-life of the L-TAG [19], (c) the difference in geographic distribution and incidence of SV40 virus strains [17], (d) an underestimation of viral content as the DNAs recovered from paraffin-embedded tissues are highly fragmented [20], and (e) on the other hand the capability of adopting the “hit and run model” for L-TAG induced transformation claiming that viruses can mediate cellular transformations through an initial “hit” while maintenance of the transformed state is compatible with the loss “run” of viral molecules [21, 22].

Moreover, few polymerase chain reaction (PCR) studies have shown the presence of SV40 in lymphomas with contradicting results. Two Egyptian studies using multiplex nested PCR have shown that SV40 DNA sequences were found in 53.8% of NHL patients in both series [23, 24]. Other studies found 13, 10, 14, 42, and 43% incidence of SV40 in NHL, respectively [17, 25, 26]. However, other studies have not supported these findings [27, 28]. Despite these contradicting results, a recent report concluded that SV40 should be added to the list of factors playing a role in the pathogenesis of B-cell lymphoma, acting together with mutated p53 in the multistep tumorigenesis of lymphoproliferative disorders [4].

The reasons for the discrepant findings are not clear. You and colleagues assumed that a fascinating possibility of some viral microRNAs (miRNAs) may function as orthologs of cellular miRNAs, but the function of most of them is unknown [29, 30]. SV40-encoded miR-S1-5p was reported to downregulate the expression of viral T-antigen without reducing the yield of infectious virus, thus reducing host cytotoxic T lymphocyte (CTL) susceptibility and local cytokine release. This dispensable downregulation appears to be very helpful in maintaining the long-term relationship between the virus and the host during latent viral infection or virus-mediated tumorigenesis [34]. Nevertheless, the orthologous role of SV40-miR-S1-5p with cellular miR423-5p also implied that SV40-encoded miRNA not only autoregulates its viral gene expression but also may regulate cellular gene expression [29].

The functions and expression of SV40 are a complex process that depends on numerous factors depending on the cellular context, virus host interaction, and accuracy and sufficiency of detection techniques. E2F is a family of transcription factors that regulate the expression of genes involved in a wide range of cellular processes [5]. In the present study, E2F1 was expressed in 5/20 (25%) of reactive follicular hyperplasia cases mainly localized to the proliferating germinal center and few in the interfollicular areas with complete negativity in the mantle zone lymphocytes. Our results agreed with other reports that stated that E2F1 is a transcription factor that mediates cell-cycle progression from the G1 to S phase and is normally regulated by a group of proteins, including cyclin D1, mainly in the germinal center [31, 32].

In the current study all DLBCL cases with high mitosis (high Ki-67 LI) had high E2F1 expression that was consistent with other studies [31, 32]. They stated that E2F1 regulates the transcription of many genes necessary for G1/S and G2/M phase transitions, DNA replication, synthesis, and mitosis [6, 31, 33].

E2F1 modulates cell death via activation of proapoptotic genes and by inactivation of antiapoptotic survival factors through p53-dependent or p53-independent pathways [34] that is consistent with the current results as DLBCL cases with numerous apoptosis belonged to high E2F1 expression.

E2F1 can stabilize p53 via transcriptional induction of p14ARF, which binds directly to mouse double minutes (MDM2) and inhibits its ability to target p53 for subsequent degradation resulting in p53 accumulation and subsequent activation of its downstream target genes required for apoptosis [6]. A second major mechanism by which E2F1 sensitizes cells to apoptosis is mediated in a p53-independent manner through antiapoptotic signaling mediated by NF*κ*B and Bcl-2 [35].

Viral T-antigens can bind all members of the pRb family promoting the activation of the E2F family, thus inducing the expression of genes required for the entry to the S phase [36]. However, the current study revealed no association between E2F1 expression and presence of SV40 was observed in DLBCL cases. This study offers novel insights into the assumed E2F1 activity that is not seemingly to have a relationship with the accused SV40 in DLBCL of the Egyptian patients.

According to the survival analysis multivariate Cox regression hazard analysis revealed that overexpression of E2F1 is independent prognostic factor for DLBCL cases studied and associated with dismal outcome.

Several reports were concordant with our findings on breast carcinoma, esophageal squamous cell carcinoma, pancreatic ductal carcinoma, non-small-cell lung cancer, and glioblastoma [37–41]. Few reports were in contrast with our findings; low E2F-1 was associated with shortened survival of DLBCL and bladder carcinoma patients [13, 42]. However, squamous cell lung carcinoma cases have no prognostic impact of E2F1 [43].

This dual role of E2F1 in cell-cycle progression and apoptosis gave it the property to be used as a target therapy. Currently, it is hypothesized that the evidence is inadequate to accept or to reject a causal relationship between SV40 and DLBCL in Egyptian patients. E2F1 has a putative oncogenic signaling in DLBCL in the current series by orchestrating and engaging cell death pathways either alone or in cooperation with cellular proliferation pathways. Overexpression of E2F1 is an indicator for short overall survival in DLBCL patients. It is therefore assumed that upregulation of E2F1 has been implicated in oncogenesis, prognosis, and prediction of therapeutic response together with development of novel target therapy. In DLBCL, the assumed E2F1 activity is not seemingly to have a relationship with the accused SV40.

## Figures and Tables

**Table 1:** Clinicopathological characteristics of DLBCL cases studied.

Variables	DLBCL (*n* = 85) No (%)
Age	
$\overset{-}{x}$ ± SD	54.31 ± 16.07
Median	56.0
Range	2.0–87.0
<60	49 (58)
≥60	36 (42)
Gender	
Male	42 (49)
Female	43 (51)
Primary site of involvement	
Nodal	56 (66)
Extranodal	29 (34)
Number of involved extranodal sites	
0	47 (55)
1-2	32 (38)
>2	6 (7)
Status	
Generalized	16 (29)
Localized	40 (71)
Size (cm)	
≤10	74 (87)
>10	11 (13)
Stage grouping	
Early	48 (56)
Advanced	37 (44)
PS	
<2	14 (16)
≥2	71 (84)
B symptoms	
Present	51 (60)
Absent	34 (40)
LDH	
Normal	11 (13)
Elevated	74 (87)
Prognostic group of R-IPI	
Good	29 (34)
Poor	56 (66)
Risk groups of AAIPI ≥60	
Low	6 (7)
Intermediate	16 (19)
High	17 (20)
Risk groups of AAIPI <60	
Low	17 (20)
Intermediate	11 (13)
High	18 (21)
Type of DLBCL	
Germinal	49 (57.6)
Nongerminal	36 (42.4)
Necrosis	
Present	14 (16)
Absent	71 (84)
$\overset{-}{x}$ ± SD	34.3 ± 22.4
Range	10.0–0.80
Mitosis	
$\overset{-}{x}$ ± SD	22.5 ± 9.5
Range	6.0–46.0
Media	21.0
Ki-67 LI	
<50	50 (59)
≥50	35 (41)
Apoptosis	
$\overset{-}{x}$ ± SD	13.5 ± 6.4
Range	3.0–31.0
Median	13.0

R-IPI: revised international prognostic index; AAIPI: age adjusted international prognostic index.

PS: performance status; LDH: lactate dehydrogenase; Ki-67 LI: Ki-67 labeling index.

DLBCL: diffuse large B-cell lymphoma.

**Table 2:** Descriptive data for SV40 expression in DLBCL.

Variables	Positive SV40 (*n* = 3) No (%)	Negative SV40 (*n* = 82) No (%)
Age		
$\overset{-}{x}$ ± SD	31.0 ± 19.5	55.2 ± 15.4
Median	30.0	56.0
Range	12–51	2–87
<60	1 (2)	48 (98)
≥60	2 (6)	34 (94)
Gender		
Male	1 (2)	41 (98)
Female	2 (5)	41 (95)
Primary site of involvement		
Nodal	2 (4)	54 (96)
Extranodal	1 (3)	28 (97)
Number of involved extranodal sites		
0	1 (2)	46 (98)
1-2	0 (0)	32 (100)
>2	2 (33)	4 (67)
Status		
Generalized	2 (12)	14 (88)
Localized	1 (2)	39 (98)
Size (cm)		
≤10	2 (3)	72 (97)
>10	1 (9)	10 (91)
Stage grouping		
Early	0 (0)	48 (100)
Advanced	3 (8)	34 (92)
PS		
<2	0 (0)	14 (100)
≥2	3 (4.2)	68 (95.8)
B symptoms		
Present	2 (4)	49 (96)
Absent	1 (3)	33 (97)
LDH		
Normal	0 (0)	11 (100)
Elevated	3 (4)	71 (96)
Prognostic group of R-IPI		
Good	0 (0)	29 (100)
Poor	3 (5)	53 (95)
Risk groups of AAIPI ≥60		
Low	0 (0)	0 (0)
Intermediate	0 (0)	0 (0)
High	2 (14)	0 (0)
Risk groups of AAIPI <60		
Low	0 (0)	0 (0)
Intermediate	0 (0)	0 (0)
High	1 (100)	0 (0)
Type of DLBCL		
Germinal	2 (4)	47 (96)
Nongerminal	1 (2.8)	35 (97.2)
Necrosis		
Present	0 (0)	14 (100)
Absent	3 (14)	68 (96)
$\overset{-}{x}$ ± SD	—	34.29 ± 22.43
Range	—	10.0–80.0
Mitosis		
$\overset{-}{x}$ ± SD	21.3 ± 1.5	22.5 ± 9.7
Range	20–23	6–46
Media	21.0	21.5
Ki-67 LI		
<50	3 (4)	74 (96)
≥50	0 (0)	8 (100)
Apoptosis		
$\overset{-}{x}$ ± SD	12.7 ± 3.1	13.5 ± 6.5
Range	10–16	3–31
Median	12	13

R-IPI: revised international prognostic index; AAIPI: age adjusted international prognostic index.

PS: performance status; LDH: lactate dehydrogenase; Ki-67 LI: Ki-67 labeling index.

DLBCL: diffuse large B-cell lymphoma.

**Table 3:** Relationship of E2F1 expression with the clinicopathological data and expression of SV40 in DLBCL cases.

Variables	E2F1 among DLBCL		Test of significance and *p* value
	≤10 (*n* = 41)	>10 (*n* = 44)	
	No (%)	No (%)	
Age			
($\overset{-}{x}$ ± SD)	58.5 ± 6.8	50.4 ± 14.5	**t** ** = 2.36** 0.020^**∗**^
Median	69.0	56	
Range	32–75	41–70	
Age			
<60	16 (33)	33 (67)	**χ** ^2^ ** = 11.25** 0.001^**∗∗**^
≥60	25 (69)	11 (31)	
Gender			
Male	21 (50)	21 (50)	**χ** ^2^ = 0.10 0.748
Female	20 (47)	23 (53)	
Primary site of involvement			
Nodal	29 (52)	27 (48)	*χ* ^2^ = 0.83 0.363
Extranodal	12 (41)	17 (59)	
Number of involved extranodal sites			
0	25 (3)	22 (47)	FE = 1.21 0.553
1-2	13 (41)	19 (59)	
>2	3 (50)	3 (50)	
Status (nodal = 56)			
Generalized	9 (40)	9 (60)	FE **=** 0.809 0.779
Localized	17 (53)	21 (47)	
Size (cm)			
≤10	36 (49)	6 (51)	**χ** ^2^ = 0.04 0.843
>10	5 (45)	38 (55)	
Stage grouping			
Early	25 (52)	23 (48)	*χ* ^2^ = 0.65 0.419
Advanced	16 (43)	21 (57)	
PS			
<2	7 (50)	7 (50)	FE = 1.088 1.000
≥2	34 (48)	37 (52)	
B symptoms			
Present	23 (45)	28 (55)	*χ* ^2^ = 0.50 0.478
Absent	18 (53)	16 (47)	
LDH			
Normal	3 (27)	8 (73)	*χ* ^2^ = 2.22 0.136
Elevated	38 (51)	36 (49)	
Prognostic group of R-IPI			
Good	14 (48)	15 (52)	*χ* ^2^ = 0.00 0.996
Poor	27 (48)	29 (52)	
Risk groups of AAIPI ≥60			
Low	12 (86)	2 (14)	FE = 3.09 0.233
Intermediate	5 (63)	3 (37)	
High	6 (55)	5 (45)	
Risk groups of AAIPI <60			
Low	2 (15)	11 (85)	**FE = 5.85** 0.049^**∗**^
Intermediate	10 (66)	8 (44)	
High	6 (29)	15 (71)	
Type of DLBCL			
Germinal	22 (45)	27 (55)	FE = 1.34 0.80
Nongerminal	18 (50)	18 (50)	
Necrosis			
Present	5 (36)	9 (64)	*χ* ^2^ = 1.05 0.305
Absent	36 (51)	35 (49)	
Necrosis (%)			
$\overset{-}{x}$ ± SD	44.0 ± 23.0	28.9 ± 21.5	*U* = 1.15 0.249
Median	60	20	
Range	10–60	10–80	
Mitosis			
$\overset{-}{x}$ ± SD	14.5 ± 4.4	29.9 ± 6.3	**U** ** = 12.98** <0.001^**∗∗**^
Median	13	34	
Range	12–14	24–43	
Ki-67 LI			
<50	41 (82)	9 (18)	**χ** ^2^ ** = 55.44** <0.001^**∗∗**^
≥50	0 (0)	35 (100)	
Apoptosis			
$\overset{-}{x}$ ± SD	10.5 ± 4.9	16.3 ± 6.4	**U** ** = 4.29** <0.001^**∗∗**^
Median	11	17	
Range	4–26	5–25	
SV40			
Positive	1 (33)	2 (67)	FE = 0.28 1.00
Negative	40 (49)	42 (51)	

R-IPI: revised international prognostic index; AAIPI: age adjusted international prognostic index; PS: performance status; LDH: lactate dehydrogenase; ^*∗*^significant; ^*∗∗*^highly significant; *t*-test: Student's *t*-test; *U*: Mann-Whitney test; FE: Fisher's exact test; *χ* ^2^: Chi-square test; Ki-67 LI: Ki-67 labeling index; DLBCL: diffuse large B-cell lymphoma.
